# An Antigenically Diverse, Representative Panel of Envelope Glycoproteins for Hepatitis C Virus Vaccine Development

**DOI:** 10.1053/j.gastro.2021.10.005

**Published:** 2022-02

**Authors:** Jordan H. Salas, Richard A. Urbanowicz, Johnathan D. Guest, Nicole Frumento, Alexis Figueroa, Kaitlyn E. Clark, Zhenyong Keck, Vanessa M. Cowton, Sarah J. Cole, Arvind H. Patel, Thomas R. Fuerst, Heidi E. Drummer, Marian Major, Alexander W. Tarr, Jonathan K. Ball, Mansun Law, Brian G. Pierce, Steven K.H. Foung, Justin R. Bailey

**Affiliations:** 1 Department of Medicine, Johns Hopkins University School of Medicine, Baltimore, Maryland; 2 School of Life Sciences, Faculty of Medicine and Health Sciences, The University of Nottingham, Nottingham, United Kingdom; 3 Wolfson Centre for Global Virus Research, The University of Nottingham, Nottingham, United Kingdom; 4 National Institute for Health Research Nottingham Biomedical Research Centre, Nottingham University Hospitals National Health Service Trust, Nottingham, United Kingdom; 5 Institute for Bioscience and Biotechnology Research, University of Maryland, Rockville, Maryland; 6 Department of Cell Biology and Molecular Genetics, University of Maryland, College Park, Maryland; 7 Department of Pathology, Stanford University School of Medicine, Stanford, California; 8 Medical Research Council–University of Glasgow Centre for Virus Research, Glasgow, United Kingdom; 9 Viral Entry and Vaccines Group, Burnet Institute, Melbourne, Victoria, Australia; 10 Department of Microbiology and Immunology, Peter Doherty Institute for Infection and Immunity, University of Melbourne, Melbourne, Victoria, Australia; 11 Department of Microbiology, Monash University, Clayton, Victoria, Australia; 12 Division of Viral Products, Center for Biologics Evaluation and Research, United States Food and Drug Administration, Silver Spring, Maryland; 13 Department of Immunology and Microbiology, The Scripps Research Institute, La Jolla, California

**Keywords:** Hepatitis C Virus, Broadly Neutralizing Antibodies, Neutralizing Breadth, Vaccine, bNAbs, broadly neutralizing antibodies, CD, cluster of differentation, CMV, cytomegalovirus, E1, envelope protein 1, E, envelope protein 2, euHCVdb, European Hepatitis C Virus Database, Gag, group-specific antigen, gt, genotype, HCV, hepatitis C virus, HCVcc, hepatitis C virus produced in cell culture, HCVpp, hepatitis C virus pseudoparticle, HEK, human embryonic kidney, HEPC, hepatitis C, IC_50_, 50% inhibitory concentrations, mAb, monoclonal antibody, MLV, murine leukemia virus, mockpps, mock pseudoparticle, PBS, phosphate-buffered saline, RLU, relative light units, UKNP, United Kingdom Nottingham Panel

## Abstract

**Background & Aims:**

Development of a prophylactic hepatitis C virus (HCV) vaccine will require accurate and reproducible measurement of neutralizing breadth of vaccine-induced antibodies. Currently available HCV panels may not adequately represent the genetic and antigenic diversity of circulating HCV strains, and the lack of standardization of these panels makes it difficult to compare neutralization results obtained in different studies. Here, we describe the selection and validation of a genetically and antigenically diverse reference panel of 15 HCV pseudoparticles (HCVpps) for neutralization assays.

**Methods:**

We chose 75 envelope (E1E2) clones to maximize representation of natural polymorphisms observed in circulating HCV isolates, and 65 of these clones generated functional HCVpps. Neutralization sensitivity of these HCVpps varied widely. HCVpps clustered into 15 distinct groups based on patterns of relative sensitivity to 7 broadly neutralizing monoclonal antibodies. We used these data to select a final panel of 15 antigenically representative HCVpps.

**Results:**

Both the 65 and 15 HCVpp panels span 4 tiers of neutralization sensitivity, and neutralizing breadth measurements for 7 broadly neutralizing monoclonal antibodies were nearly equivalent using either panel. Differences in neutralization sensitivity between HCVpps were independent of genetic distances between E1E2 clones.

**Conclusions:**

Neutralizing breadth of HCV antibodies should be defined using viruses spanning multiple tiers of neutralization sensitivity rather than panels selected solely for genetic diversity. We propose that this multitier reference panel could be adopted as a standard for the measurement of neutralizing antibody potency and breadth, facilitating meaningful comparisons of neutralization results from vaccine studies in different laboratories.

## Graphical abstract

**Figure:**
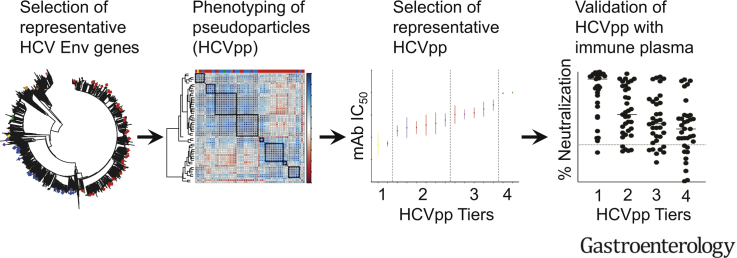

## Teaser

A panel of hepatitis C virus pseudoparticles with 4 tiers of antibody resistance was developed. These pseudoparticles can be used to measure neutralizing breadth of antibodies induced by candidate vaccines.

See editorial on page 396.

**What You Need to Know**

**Background And Context**

Development of a prophylactic hepatitis C virus vaccine will require accurate measurement of neutralizing breadth of vaccine-induced antibodies.

**New Findings**

Genetic and phenotypic analyses were used to select a panel of 15 representative hepatitis C virus pseudoparticles spanning 4 tiers of neutralization resistance.

**Limitations**

Most of the hepatitis C virus pseudoparticles in the panel were derived from genotype 1 isolates, so the panel may not fully represent all isolates from genotypes 2 through 8.

**Impact**

This reference panel could be adopted as a standard for measurement of neutralizing antibody potency and breadth, facilitating comparisons of neutralization results from vaccine trials.

Direct-acting antiviral therapies for hepatitis C virus (HCV) infection represent a major advancement toward reducing the global burden of liver disease.^1^ However, due to limited uptake of treatment and the high incidence of new infections, few countries are on target to achieve the elimination of HCV as a public health problem by 2030, a goal set by the World Health Organization.^2^ Therefore, an effective prophylactic vaccine is needed to advance efforts toward HCV elimination.^3^^,^^4^ HCV prevention with a prophylactic vaccine is also important to prevent HCV-induced hepatocellular carcinoma, because the risk of hepatocellular carcinoma persists in some patients even after successful treatment with direct-acting antiviral therapies.^5^^,^^6^

The extreme genetic diversity of HCV is a barrier to vaccine development,^7^^,^^8^ but some broadly neutralizing antibodies (bNAbs) recognize relatively conserved envelope glycoprotein (E1E2) domains and block infection by genetically diverse HCV isolates.^9^, ^10^, ^11^, ^12^, ^13^, ^14^, ^15^, ^16^ Development of bNAbs is associated with natural control of HCV infection in some humans, and bNAbs can prevent HCV infection in animal models.^9^^,^^13^^,^^17^, ^18^, ^19^, ^20^, ^21^, ^22^ Thus, an effective HCV vaccine will certainly need to induce bNAbs or a broadly neutralizing polyclonal antibody response. Therefore, accurate and standardized measurement of neutralizing breadth of antibodies is critical to guide vaccine development.

Antibody neutralizing breadth is typically measured using panels of HCV pseudoparticles (HCVpps) or replication-competent cell culture viruses (HCVccs). HCVpps are lentiviral particles with HCV E1E2 proteins on their surface, which enable the measurement of single rounds of viral entry into hepatoma cells.^23^, ^24^, ^25^ Despite structural differences between HCVccs and HCVpps,^26^^,^^27^ multiple studies have demonstrated concordance between neutralization results of identical E1E2 clones expressed in HCVpps or HCVccs,^28^, ^29^, ^30^, ^31^ suggesting that either approach can be used to measure antibody neutralizing activity in vitro.

The HCVpp and HCVcc panels used until now to measure neutralizing breadth have notable limitations. Our previous panel of 81 patient-derived clones was tested against a limited set of cluster of differentiation (CD) 81 binding site monoclonal antibodies (mAbs).^29^ Most other panels are relatively small and do not represent the polymorphisms present in naturally circulating HCV isolates.^32^, ^33^, ^34^ In addition, these panels were generally assembled with an emphasis on genetic rather than antigenic diversity, and they have not been evaluated with a standard set of neutralizing antibodies or immune sera to define the range of neutralization sensitivity of isolates in each panel. Some panels may contain only neutralization-sensitive or neutralization-resistant isolates. Lack of standardization makes it difficult to compare neutralization results obtained in different studies.

Here, we describe the selection and validation of a genetically and antigenically diverse reference panel of 15 HCVpps for use in neutralization assays. These HCVpps were selected to maximize the representation of natural polymorphisms observed in HCV sequences that are available in international sequence databases, with a focus on genotype 1 because infections with this genotype are most prevalent worldwide.^35^ In addition, we selected antigenically diverse HCVpps that demonstrated a wide range of neutralization sensitivity to diverse broadly neutralizing mAbs and human plasma. We propose that this reference panel could be adopted as a standard for the measurement of antibody neutralizing potency and breadth to enable comparisons of neutralization results from different laboratories.

## Materials and Methods

### Cell Lines

A single source of both wild-type human embryonic kidney (HEK) 293T and Huh7 human hepatoma cell lines^29^ was used. As indicated, CD81-knockout HEK293T cells^36^ (Dr Joe Grove, University of Glasgow, Glasgow, United Kingdom) were used for production of some HCVpps (Supplementary Figure 1). Cells were grown in Dulbecco’s Modified Essential Medium (Invitrogen, Carlsbad, CA) supplemented with 10% fetal bovine serum (FBS) and 0.1 mmol/L nonessential amino acids (Invitrogen).

### Antibodies

HCV mAbs CBH-7,^11^ HC84.26,^10^ HC33.4, and HC33.1,^12^ and negative control mAb R04 to cytomegalovirus (CMV) were produced by Steven Foung. mAbs AR3A^9^ and AR4A^13^ were produced by Mansun Law. mAb hAP33 (a chimeric mouse AP33-human Fc antibody)^16^^,^^37^ was produced by Arvind Patel, and mAb HCV1^38^ was a kind gift of Yang Wang (MassBiologics, Boston, MA). mAbs hepatitis C (HEPC)74, HEPC98, HEPC108, HEPC111, HEPC112, and HEPC146 were a kind gift of James E. Crowe Jr (Vanderbilt University Medical Center, Nashville, TN).^15^^,^^39^

### Plasma

Plasma from genotype 1- to 3-infected donors was obtained from the Baltimore Before and After Acute Study of Hepatitis (BBAASH).^40^ Plasma samples representing genotype 4 to 6 HCV infections were obtained from the University of Nottingham Trent HCV Cohort study.^41^ Plasma was heat inactivated at 56°C for 30 minutes before use. All participants provided written informed consent for blood donation, and protocols were approved by the Johns Hopkins University School of Medicine Institutional Review Board or the Northern & Yorkshire Multicentre Research Ethics Committee (ref. MREC/98/3/55).

### Sequence Analysis

Genotype 1 to 7 HCV E1E2 amino acid sequences were downloaded from the National Center for Biotechnology Information (NCBI; www.ncbi.nlm.nih.gov), the European Hepatitis C Virus database (euHCVdb),^42^ and Los Alamos National Laboratory HCV^43^ databases. Redundant or incomplete sequences were removed. These sequences and E1E2 sequences from 2 previously published HCVpp panels^28^^,^^29^ (166 sequences) were aligned using Multiple Alignment using Fast Fourier Transform (MAFFT) 7.3 software.^44^ Pairwise sequence distance matrices were calculated from the multiple sequence alignment, which were input to R software (www.r-project.org) to perform hierarchical phylogenetic clustering. For each candidate panel of size n, the hierarchical clustering cutoff was specified to generate n clusters, and candidate sequence panels were identified by selecting 1 exemplar sequence for each cluster. Polymorphism coverage for each candidate panel was calculated using the multiple sequence alignment and an in-house Perl script. GenBank accession numbers for the 75 E1E2 clones used for HCVpp production are included in Supplementary Table 1. E1E2 expression plasmids for the final panel of 15 HCVpps are available from Addgene (www.addgene.org).

### Hepatitis C Virus Pseudoparticles Production

HIV group-specific antigen (Gag)-packaged HCVpps were generated by lipofectamine-mediated transfection of HCV E1E2 plasmid, pNL4-3.Luc.R-E-plasmid containing the *env*-defective HIV proviral genome (National Institutes of Health AIDS Reagent Program), and pAdVantage (Promega, Madison, WI) plasmid into HEK293T cells, as described.^45^ Murine leukemia virus (MLV) Gag-packaged HCVpps were produced by polyethylenimine (Polyscience) mediated transfection of HCV E1E2 plasmid, luciferase-encoding reporter plasmid (pTG126), and phCMV MLV Gag/polymerase packaging construct (phCMV-5349) plasmid into HEK293T cells, as described.^45^ Mock pseudoparticles (mockpps) generated without E1E2 plasmid were used as a negative control for each transfection. CD81-knockout HEK293T cells were used for production of HCVpps used for testing of genotype 2 and 3 sera and for production of HCVpps used for E2 quantitation. As previously shown,^36^ HCVpps produced in CD81-knockout HEK293T cells consistently showed greater entry of hepatoma cells, but neutralization results obtained using HCVpps produced in wild-type or CD81-knockout HEK293T cells were highly correlated (Supplementary Figure 2).

### Hepatitis C Virus Pseudoparticles Entry

Huh7 cells (15,000 per well) were plated in 96-well microplates and incubated overnight. Then, 50 μL of HCVpps were added to the Huh7 cells in triplicate, and plates were incubated at 37°C for 5 hours. HCVpps were removed and replaced with phenol-free media, and cells were incubated for 72 hours at 37°C. HCVpps entry was determined by measurement of luciferase activity of cell lysate in relative light units (RLU). Sixty genotype 1a and 1b HIV Gag-packaged HCVpps showed greater specific entry than MLV-Gag HCVpps (Supplementary Figure 3), so the HIV-Gag production protocol was used to produce HCVpps for neutralization testing.

### Neutralization

Neutralization assays were performed as described previously.^45^ mAbs were serially 5-fold diluted, starting at a concentration of 100 μg/mL (leaving the last well as phosphate-buffered saline [PBS] only), and incubated with HCVpps for 1 hour at 37°C before addition to Huh7 target cells in duplicate. HCVpps entry was measured as above. The percentage of neutralization was calculated as [1 − (RLU_mAb_/RLU_PBS_)] × 100, with the PBS RLU values averaged across 3 plates. R04 and polyclonal human IgG (Thermo Fisher, Waltham, MA) were used as negative controls. Log_10_ 50% inhibitory concentrations (log_10_IC_50_) were calculated from neutralization curves fit by nonlinear regression (log[inhibitor] vs normalized response, variable slope) in Prism 8 software (GraphPad Software, San Diego, CA). mAb-HCVpp tests that did not reach 50% inhibition were assigned an IC_50_ of 100 μg/mL. IC_50_ values for 7 mAbs generated with the final panel of 15 HCVpps are listed in Supplementary Table 2. Plasma samples were tested at a 1:20 dilution. Pooled plasma from 10 HCV-negative donors also at 1:20 dilution was used as a negative control. The percentage neutralization of each HCVpp was calculated as [1 − (RLU_immune plasma_/RLU_control plasma_)] × 100.

### Hierarchical Clustering

The log_10_IC_50_ values for each of 7 mAbs (with HCV1 and AP33 values averaged together) for each HCVpp were compared pairwise for all HCVpps using Pearson’s correlation, as described in Results. Rho (ρ) values were used as input for hierarchical clustering as implemented in the “pvclust” package for R software. This clustering, depicted as a tree, was also used to order a matrix of correlation values produced using the “corrplot” package for R software.

### E2 Quantitation

HCVpps were concentrated 30-fold using a 100-kDa molecular weight cutoff (Thermo Fisher) and run on 4% to 12% denaturing, reducing 2,2-Bis(hydroxymethyl)-2,2′2′′-nitrilotriethanol (BIS)-Tris gels. Proteins were visualized with mAbs HC33.1 and anti–HIV-1 p24 (cat: 9044l; Abcam, Cambridge, MA), horseradish peroxidase-conjugated secondary antibody, and SuperSignal West Femto Maximum Sensitivity Substrate (Thermo Fisher), and imaged on the ChemiDoc XRS+ (Bio-Rad, Hercules, CA). For enzyme-linked immunosorbent assay, HCVpps were diluted 1:10 in Tris-buffered saline with 0.5% bovine serum albumin, 1.0% sodium dodecyl sulfate, and 50 nmol/L dithiothreitol, and boiled at 100°C, serially diluted 1:2, and then incubated in *Galanthus nivalis* lectin–coated microwells. Wells were washed and incubated with HC33.1 or IgG (negative control), followed by anti-human IgG horseradish peroxidase–conjugated secondary antibody, and 3,3′,5,5′′-tetramethylbenzidine substrate.

## Results

### Selection of Hepatitis C Virus Envelope Glycoprotein 1 and 2 Clones for Hepatitis C Virus Pseudoparticles Production

**Figure 1:**
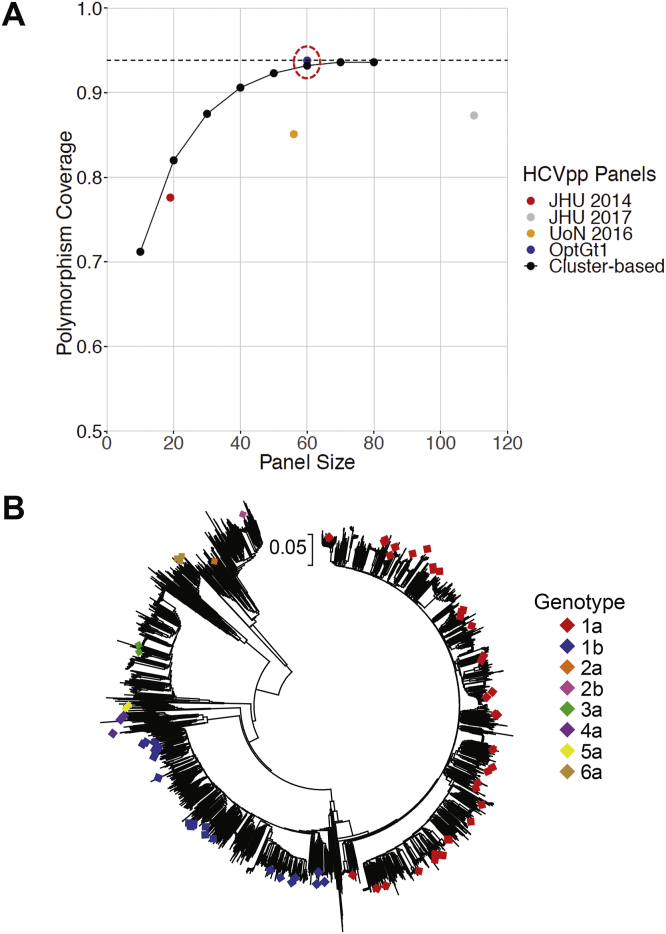
Selection of genetically diverse and representative E1E2 strains for HCVpp phenotyping. (*A*) Percentage representation by candidate panels of polymorphisms observed with 2% to 80% frequency in a set of 2587 nonredundant gt1 E1E2 sequences. Polymorphism coverage by a set of 166 functional genotype 1 E1E2 clones is shown as a *black dotted line* (93.8%). Polymorphism coverage by previously published panels of 19 genotype 1 HCVpps (John Hopkins University [JHU] 2014), 113 genotype 1 HCVpps (JHU 2017),^28^ and 58 genotype 1 HCVpps (University of Nottingham [UoN] 2016),^29^ are indicated. Candidate genotype 1 E1E2 panels of various sizes, selected by hierarchical phylogenetic clustering (cluster-based), are shown as *black points*, and coverage by an optimized panel of 60 genotype 1 E1E2 clones (OptGt1) is shown as a *blue point circled in red* (93.8%). (B) Neighbor-joining phylogenetic tree of 3583 E1E2 amino acid sequences representing all subtypes of genotypes 1 to 7. The tree was generated in Molecular Evolutionary Genetics Analysis (MEGA) 7.0.21 software using the Jones-Taylor-Thornton model with gamma distribution. The *branches* are drawn to scale, and positions containing gaps and missing data were eliminated. The *symbols* indicate 75 genotype 1 to 6 clones selected for HCVpp production.

We downloaded all available genotype 1 to 7 E1E2 sequences from the NCBI GenBank, Los Alamos National Laboratory,^43^ and euHCVdb^42^ databases, including 2587 genotype 1 (77%) and 830 genotype 2 to 7 (23%) sequences. We focused our initial polymorphism analysis on genotype 1 sequences, because genotype 1 infection is most prevalent worldwide and is best represented in sequence databases. Using the aligned set of 2587 genotype 1 E1E2 amino acid sequences, we developed a script to identify all possible amino acid polymorphisms appearing at each position in E1E2. To focus on common variations and ignore universally conserved positions, we only tabulated polymorphisms appearing in 2% to 80% of database isolates. Because the functional status of most of the sequence database E1E2 isolates is unknown, we assessed representation of these database sequence polymorphisms by a known functional set of 166 genotype 1a and 1b E1E2 clones.^28^^,^^29^ The entire set of 166 functional clones contained 93.8% of amino acid polymorphisms present in 2% to 80% of genotype 1 database sequences. Interestingly, we found that we could identify a subset of 60 functional E1E2 clones that contained as many database polymorphisms (93.8%) as the full 166 functional E1E2 panel (Figure 1*A*).^28^^,^^29^ Because the inclusion of additional clones added no additional polymorphism coverage, we limited subsequent genotype 1 phenotyping to these 60 E1E2 clones.

HCVpps produced with 3 of these clones (1a18, United Kingdom Nottingham Panel [UKNP]1.21.4, and UKNP1.21.5) were poorly functional in initial tests (Supplementary Figure 3), so replacement clones genetically similar to each were added to the phenotyping set (UKNP1.2.5, UKNP1.21.2, and UKNP1.21.3, respectively). In addition, we selected 12 genotype 2 to 6 E1E2 clones from among the limited set of functional clones that had been previously described, choosing clones previously shown to be highly neutralization sensitive or highly resistant.^29^ The 75 E1E2 clones selected for phenotypic analysis were widely distributed across clades in a phylogenetic tree of 3583 genotype 1 to 7 sequences from all subtypes (Figure 1*B*).

### Wide Variation in Hepatoma Cell Entry of Hepatitis C Virus Pseudoparticles

**Figure 2:**
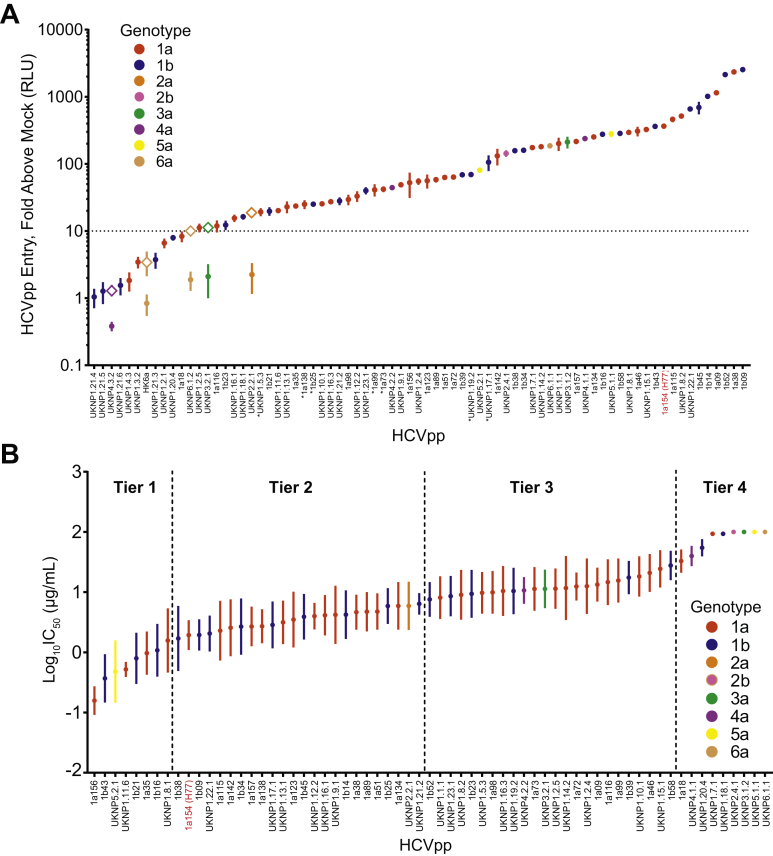
Wide variation in hepatoma cell entry and neutralization sensitivity of HCVpp. (*A*) Hepatoma cell entry of 75 HCVpps expressed as fold increase in RLUs compared with the background entry of mockpps. In some cases, HCVpps were first generated using HIV Gag (*circles*) and then MLV Gag (*open diamonds*). Data represent 1 to 5 independent experiments for each HCVpp, with each experiment performed with 2 to 6 replicates. A threshold of 10-fold above mockpp entry is indicated with a *dotted line*. The *asterisks* indicate HCVpps that were freeze-thawed before testing. (*B*) Neutralization of 65 HCVpps by 7 bNAbs targeting diverse epitopes across E1E2. mAb/HCVpp tests that did not reach 50% inhibition were assigned an IC_50_ of 100 μg/mL. HCVpps are arranged from most to least neutralization sensitive based on mean log_10_IC_50_ measured for 7 mAbs and divided into 4 tiers of sensitivity based on the normal distribution of the data. Each mAb-HCVpp combination was tested with 9 serial mAb dilutions in duplicate. The *points* indicate means and the *whiskers* indicate standard error of the mean. Prototype reference strain H77 is highlighted in *red*.

We measured HCVpps entry into Huh7 hepatoma cells to determine the relative function of each of the 75 E1E2 clones (Figure 2*A*). Mockpps lacking E1E2 were produced and tested in parallel with HCVpps to quantitate nonspecific entry. Specific entry of each HCVpp was calculated as a ratio of HCVpps entry relative to mockpps entry. Genotype 2 to 6 E1E2 clones that did not produce functional HCVpps using the HIV-Gag production system were then produced using an MLV-Gag production system. HCVpps demonstrated a wide range of entry (0.38- to 2532-fold greater than mockpps). Of 75 HCVpps, 63 (84%) demonstrated entry greater than the preselected threshold of 10-fold above mockpps entry using the HIV-Gag production system, whereas 2 HCVpps (clones UKNP2.2.1 and UKNP3.2.1) exceeded this threshold only when generated using the MLV-Gag HCVpps production system. Of 75 HCVpps, 10 (13%) failed to exceed the 10-fold above mockpp threshold when produced using the HIV-Gag or the MLV-Gag production systems. HCVpps expressing E1E2 from genotypes 2 to 6 were distributed across the ranking of genotype 1 HCVpp entry. The 65 HCVpps that exceeded the 10-fold above mockpps entry threshold (genotype 1, n = 56; genotype 2 to 6, n = 9) carried 96.1% of genotype 1 polymorphisms and 84.8% of genotype 2 to 6 polymorphisms present in 2% to 80% of database sequences. These 65 HCVpps were used for subsequent neutralization testing.

### Wide Variation in Neutralization Sensitivity of Hepatitis C Virus Pseudoparticles

We measured neutralization of each of the 65 HCVpps by serial dilutions of 7 well-characterized neutralizing mAbs, which were selected because they bind to a range of neutralizing epitopes across the E2 glycoprotein or E1E2 heterodimer, with a range of neutralizing breadth previously documented using other HCVpp or HCVcc panels.^9^^,^^13^^,^^16^^,^^38^ These 7 mAbs recognize 5 distinct antigenic sites, including domain B/AR3 (mAb AR3A), domain C (mAb CBH-7), domain D (mAb HC84.26), AR4 (mAb AR4A), and domain E/AS412 (HC33.4, HCV1, and hAP33). All mAbs were isolated from HCV-infected humans, except HCV1, which was generated by immunizing a transgenic mouse expressing human antibody genes,^38^ and hAP33, which was generated by immunization of a wild-type mouse,^16^ and then subsequently produced as a mouse-human chimera (ie, its variable heavy and variable light chains grafted onto a human IgG1 Fc backbone).^37^

The 65 HCVpps displayed a wide range of neutralization sensitivity to the panel of 7 mAbs. We ranked each HCVpp from lowest to highest mean log_10_IC_50_ across 7 mAbs (mean log_10_IC_50_ of −0.80 to >2 μg/mL) (Figure 2*B*). Given high similarity between epitopes and neutralization profiles of mAbs HCV1 and AP33 (Supplementary Figure 4), log_10_IC_50_ values for these 2 mAbs were averaged, giving each mAb half the weight of the other 5 mAbs in this analysis. Based on the normal distribution of these mean log_10_IC_50_ values, we separated the HCVpps into 4 tiers:

• Tier 1: 8 HCVpps with mean log_10_IC_50_ values more than 1 standard deviation below the overall mean (log_10_IC_50_ < 0.22 μg/mL)
• Tier 2: 24 HCVpps within 1 standard deviation below the mean (log_10_IC_50_ 0.22–0.83 μg/mL)
• Tier 3: 24 HCVpps within 1 standard deviation above the mean (log_10_IC_50_ 0.83–1.45 μg/mL)
• Tier 4: 9 HCVpps more than 1 standard deviation above the mean (log_10_IC_50_ > 1.45 μg/mL).

In agreement with prior studies.^17^^,^^29^ some HCVpps generated using E1E2 clones from the same subtype differed dramatically in their sensitivity to neutralization, whereas many HCVpps generated using E1E2 clones from different subtypes or even different genotypes demonstrated very similar neutralization sensitivity. As expected based on prior studies,^33^ HCVpp 1a154 (strain H77) was relatively sensitive to neutralization, falling in tier 2. Within or across subtypes, the genetic distance between E1E2 clones did not correlate with the difference in neutralization sensitivity of HCVpps produced from those clones (Supplementary Figure 5*A*). There was also no correlation between the magnitude of hepatoma cell entry of each HCVpp and the relative neutralization sensitivity of that HCVpp (Supplementary Figure 5*B*). It is noteworthy that some of the most neutralization-sensitive and neutralization-resistant HCVpps were genotype 2 to 6 strains, although this was not surprising because these E1E2 clones were selected in part based on prior testing showing them to be highly neutralization sensitive or resistant.^29^

### Hierarchical Clustering Based on Patterns of Relative Neutralization Sensitivity Reveals Antigenic Relationships Among Hepatitis C Virus Pseudoparticles

**Figure 3:**
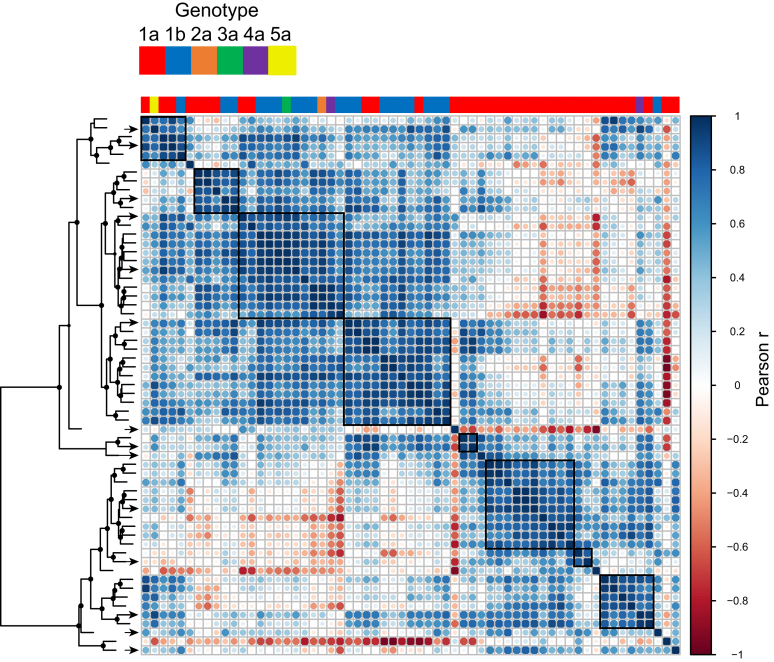
Hierarchical clustering based on patterns of relative neutralization sensitivity reveals antigenic relationships among HCVpps. The heat map shows all pairwise correlations among neutralization profiles of 61 HCVpps (neutralization profile = log_10_IC_50_ values for 7 mAbs). HCVpps are arrayed in the same order along the *x-* and *y-*axes, with the genotype of each HCVpp indicated on the *x*-axis. The *circles* at each intersection are scaled by the magnitude of Pearson’s correlation (*r*) between neutralization profiles. Hierarchical clustering analysis using these pairwise correlations is depicted as a tree. The *circles* at tree nodes indicate approximately unbiased (arbitrary unit [AU)] test values > 70, indicating strength of support for a particular cluster. HCVpps were assigned to the same antigenic group (*black boxes*) if they clustered with approximately unbiased (AU) values > 70, with mean *r* > 0.81 for correlations among the group, corresponding to *P* < .05. The *arrows* indicate HCVpps selected for the final 15 HCVpp panel.

HCVpps can be ranked by overall neutralization sensitivity to a set of mAbs, but they also have unique patterns of relative sensitivity to individual mAbs targeting different neutralizing epitopes. For example, while 2 HCVpps might have equivalent mean IC_50_ values for a set of mAbs, 1 HCVpp might be most sensitive to mAb AR3A and most resistant to mAb AR4A, whereas another is most sensitive to AR4A and most resistant to AR3A. We defined the neutralization profile for each HCVpp as a set of 6 log_10_IC_50_ values (log_10_IC_50_s for AR3A, CBH-7, HC84.26, AR4A, HC33.4, and the average log_10_IC_50_ of HCV1 and hAP33). We performed hierarchical clustering of HCVpps based on pairwise Pearson’s correlations between their neutralization profiles (Figure 3). This analysis excluded 4 HCVpps that were fully resistant to all reference mAbs (IC_50_ > 100 μg/mL). HCVpps were assigned to the same antigenic group if they clustered with approximately unbiased (arbitrary unit) values >70, with mean *r* > 0.81 for the group, corresponding to *P* < .05.

Based on clustering analysis, we found 61 HCVpps fell in 15 distinct antigenic groups, with each group containing from 1 to 12 HCVpps. Notably, clustering was not driven by genotype or subtype. Six groups contained HCVpps from multiple subtypes, and 9 groups contained only genotype 1a HCVpps, which was expected by chance given the large number of genotype 1a HCVpps included in the analysis. Of genotype 1a HCVpps, 16 (44%) fell in multi-subtype groups, whereas 20 (56%) fell in 1a-only groups (*P* = .48 by Fisher’s exact test). In some cases, neutralization profiles of genotype 2 to 6 HCVpps were highly correlated with profiles of 1 or more genotype 1 HCVpps (eg, UKNP4.1.1 (4a) and UKNP1.20.4 (1b); *r* = 0.94, *P* = .006). Overall, this analysis showed that a large number of genetically diverse HCVpps could be clustered based on neutralization profiles into a relatively small number of antigenic groups that were not dictated by genotype or subtype.

### Identification of an Antigenically and Genetically Diverse and Representative Subset of 15 Hepatitis C Virus Pseudoparticles

For ease of use and to limit redundancy, we selected a representative subset of the larger panel of 65 HCVpps based on 5 criteria. First, we selected HCVpps that would preserve the same distribution of overall neutralization sensitivity across tiers 1 to 4 that we observed with the full panel of 65 HCVpps.

Second, we selected HCVpps that would best preserve the log_10_IC_50_ mean, range, and standard deviation observed with the panel of 65 HCVpps for each of the 7 reference mAbs.

Third, we selected HCVpps to maximize the representation of the 15 hierarchical antigenic clusters.

Fourth, we included HCVpps from multiple genotypes and subtypes to maintain genetic diversity.

Fifth, we selected HCVpps that demonstrated robust hepatoma cell entry after production using the HIV-Gag HCVpp protocol (to maximize ease of use and reproducibility).

**Figure 4:**
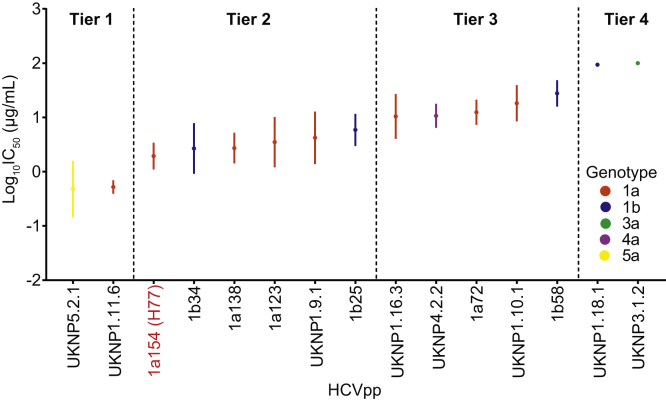
Identification of an antigenically and genetically diverse and representative subset of 15 HCVpps. HCVpps selected as representative of the larger HCVpp panel based on the distribution of neutralization sensitivity across tiers 1 to 4, preservation of the log_10_IC_50_ mean, range, and standard deviation for each of the 7 reference mAbs, representation of hierarchical antigenic clusters, inclusion of multiple genotypes and subtypes, and robust hepatoma cell entry. The *points* indicate means, and the *whiskers* indicate standard error of the mean. Prototype reference strain H77 is highlighted in *red*.

By satisfying these 5 criteria, we identified 15 HCVpps that were robustly functional and antigenically representative of the larger panel of 65 HCVpps (Figure 4). Notably, the prototype reference strain H77 (HCVpp 1a154) was included in tier 2 of this final panel.

Overall neutralization sensitivity of the panels of 65 and 15 HCVpps was very similar, mean IC_50_ of 37.80 μg/mL (range, 0.41 to >100 μg/mL) vs 35.94 μg/mL (range, 0.63 to >100 μg/mL). Like the larger panel, the panel of 15 HCVpps could be distributed across 4 tiers of overall neutralization resistance, with 2 HCVpps (13.5%) in tier 1, 6 HCVpps (40%) in tier 2, 5 HCVpps (33%) in tier 3, and 2 HCVpps (13.5%) in tier 4 (Figure 4). In addition, the neutralizing breadth (percentage of the panel neutralized) for each mAb at IC_50_ thresholds of 100, 10, or 1 μg/mL was very similar when quantitated using the panel of 65 or the panel of 15 HCVpps (Table 1). Interestingly, these 15 HCVpps were highly antigenically representative of the larger panel of 65 HCVpps despite expressing only 79.6% of genotype 1 polymorphisms and 60.3% of genotype 2 to 6 polymorphisms present in 2% to 80% of database sequences, indicating that many E1E2 amino acid differences across strains and genotypes are irrelevant for bNAb sensitivity.

**Table 1:** Neutralizing Breadth of Reference Monoclonal Antibodies Measured Using 65 or 15 Hepatitis C Virus Pseudoparticle Panels

mAbs	HCVpp No.	Neutralizing breadth,^a^*%*		
		100 μg/mL	10 μg/mL	1 μg/mL
hAP33	65	91	82	46
	15	93	80	47
HCV1	65	89	80	28
	15	87	87	40
AR4A	65	91	80	20
	15	87	80	33
HC84.26	65	74	57	34
	15	73	67	33
AR3A	65	74	54	18
	15	60	53	13
HC33.4	65	77	48	20
	15	73	40	27
CBH-7	65	31	12	3
	15	33	13	13

a Percentage of isolates in 65 or 15 HCVpp panels neutralized with IC_50_ <100, 10, or 1 μg/mL.

To better understand the differences in hepatoma cell entry and neutralization sensitivity between these 15 HCVpps, we used Western blot and enzyme-linked immunosorbent assay (Supplementary Figure 1*A* and *B*) to measure incorporation of E2 into each HCVpp. We found that incorporation of E2 varied greatly across HCVpps and that the level of E2 incorporation correlated significantly with the level of hepatoma cell entry of each HCVpp (Supplementary Figure 1*C*). However, the level of E2 incorporation did not correlate with HCVpp neutralization sensitivity (Supplementary Figure 1*D*), indicating that differences in sensitivity between HCVpps are not dictated by these differences in E2 incorporation.

### Panel Validation Using Human Immune Plasma Samples and Additional Neutralizing Monoclonal Antibodies

**Figure 5:**
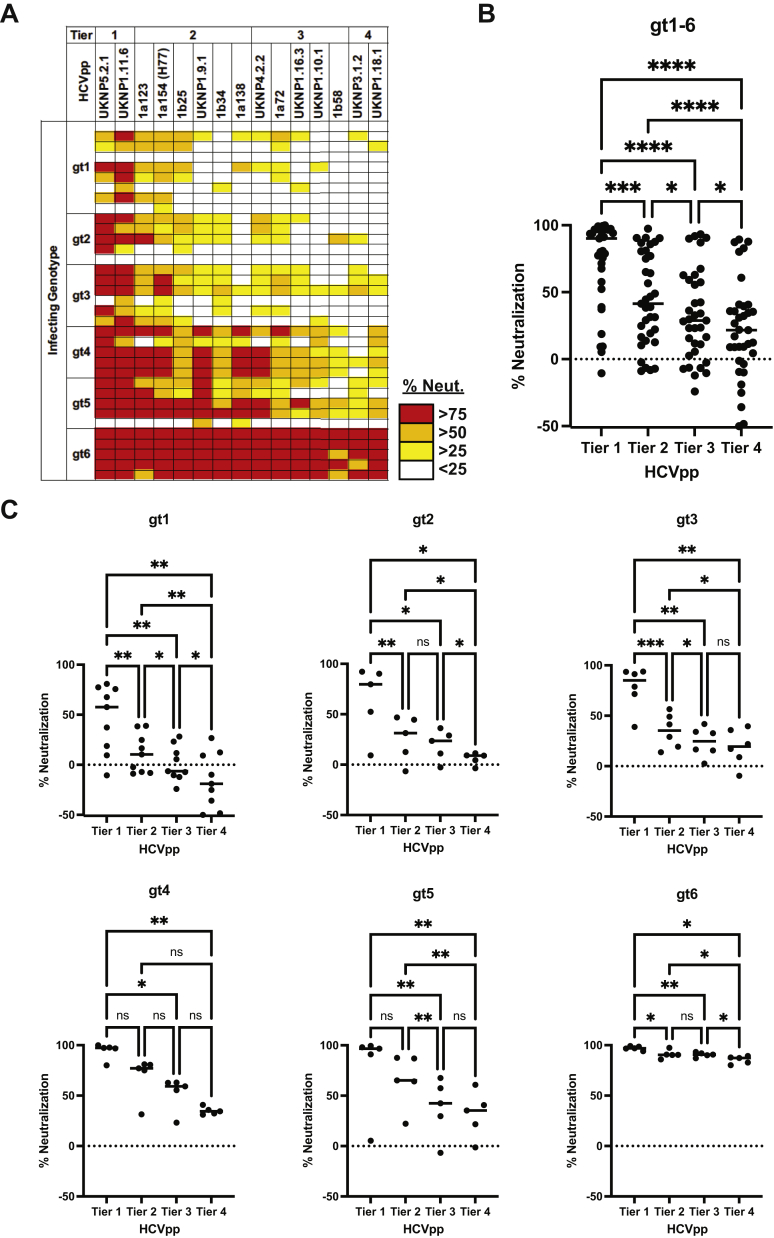
Panel validation using human immune plasma samples. (*A*) Percentage neutralization of the 15 HCVpp panel (tiers 1–4) by plasma samples at 1:20 dilution from 35 individuals infected with genotype (gt) 1 to 6 HCV, measured in duplicate. (*B* and *C*) Plasma neutralization is arranged according to HCVpp tier. Each *point* indicates mean neutralization of all HCVpps in the indicated tier by a single plasma sample from panel *A*. All plasma samples (gt1–6) were analyzed together in *B*, or separated by infecting HCV genotype in *C*. The *horizontal lines* are medians. Groups were compared by 1-way analysis of variance if data were normally distributed (gt1, 2, 3, 5, and 6 graphs) or by Friedman’s test if data were not normally distributed (gt1–6, gt4 graphs). All tests were adjusted for multiple comparisons using the Benjamini, Krieger, and Yekutieli method, with adjusted *P* < .05 considered significant. ns, not significant; ∗*P* < .05, ∗∗*P* < .005, ∗∗∗*P* < .001, ∗∗∗∗*P* < .0001.

We selected 35 plasma samples from HCV-infected humans for neutralization breadth testing using the 15 HCVpp panel. The samples were obtained from individuals infected with genotype 1 (n = 9), 2 (n = 5), 3 (n = 6), 4 (n = 5), 5 (n = 5), or 6 (n = 5). Most of the samples were obtained from individuals with chronic infection, and samples were not matched for duration of infection. As expected, these samples displayed a range of neutralizing breadth when tested at a 1:20 plasma dilution (0%–100% of the panel neutralized) (Figure 5*A* and Supplementary Table 3). Interestingly, some plasma samples with moderate or poor neutralizing breadth enhanced rather than inhibited entry of some HCVpps, which was demonstrated by greater entry of HCVpps incubated with immune plasma relative to entry of HCVpps incubated with HCV-negative control plasma, as has been previously described (Supplementary Table 3).^34^^,^^46^^,^^47^ For unclear reasons, genotype 4- to 6-infected plasma displayed greater neutralizing breadth than genotype 1- to 3-infected plasma (median 87% vs 27% of the panel neutralized). However, across all genotype 1 to 6 plasma samples, tier 1 HCVpps were significantly more sensitive to plasma neutralization than tiers 2, 3, and 4 (*P* < .001 for each comparison). Tier 2 HCVpps were significantly more sensitive than tiers 3 (*P* < .05) and 4 (*P* < .0001). Tier 3 HCVpps were significantly more sensitive than tier 4 (*P* < .05) (Figure 5*B*). Notably, this trend of increasing neutralization resistance from tiers 1 to 4 was also consistent when genotype 1- to 6-infected plasma samples were segregated by genotype, although not all comparisons between tiers were statistically significant, likely due to smaller numbers of samples in each group (Figure 5*C*).

Seven additional neutralizing mAbs (HEPC74, HEPC98, HEPC108, HEPC111, HEPC112, HEPC146, and HC33.1) were also tested using the 15 HCVpp panel because they bind to unique epitopes relative to the 7 reference mAbs used to select the panel. Four of these mAbs (HEPC98, HEPC108, HEPC112, and HEPC146) target distinct antigenic sites relative to the initial mAb reference panel.^29^^,^^39^ As expected, the mAbs showed a wide range of neutralizing breadth when tested at 20 μg/mL concentration (62% or 5% of the panel neutralized by HEPC74 or HEPC112, respectively) (Supplementary Figure 6*A*). We expected that neutralization of the panel by HEPC74 would be similar to neutralization we had observed with AR3A, given structural analyses demonstrating that the 2 mAbs bind to highly similar epitopes. This was confirmed, as the percentage neutralization of each HCVpp by HEPC74 was highly correlated with values obtained with AR3A (*r* = 0.67, *P* = .008) but not with unrelated mAb HC33.4 (*r* = 0.33, *P* = .23) (Supplementary Figure 6*B*).

Taken together, these data confirmed that immune plasma samples and novel mAbs displayed a wide range of neutralizing breadth across the HCVpp panel. Regardless of the infecting genotype of the immune plasma source, Tier 1 HCVpps were most sensitive to neutralization, followed by tier 2, tier 3, and then tier 4.

### Panel Validation by Independent Neutralization Testing in a Second Research Laboratory

**Figure 6:**
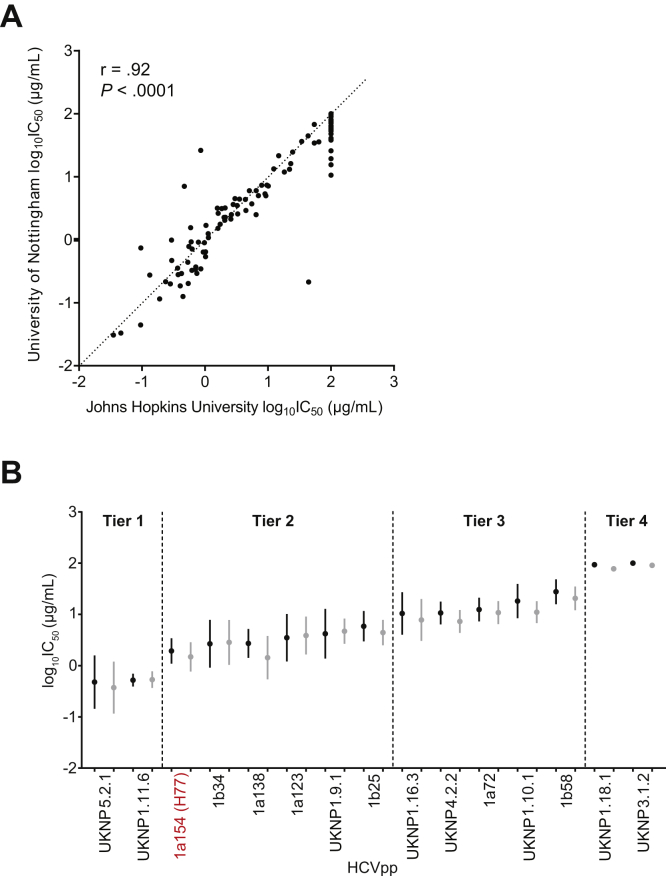
Panel validation by independent neutralization testing in a second research laboratory. Repeat production of the HCVpp panel and neutralization testing with 7 reference mAbs tested with serial dilutions in duplicate. (*A*) Correlation between log_10_IC_50_ values for each HCVpp-mAb combination obtained at Johns Hopkins or the University of Nottingham. The *r* and *P* values from Pearson’s correlation. (*B*) For each HCVpp, log_10_IC_50_ results for 7 mAbs obtained at Johns Hopkins are on the left (*black*) and repeat results obtained at the University of Nottingham are on the right (*gray*). The *points* indicate means and *whiskers* standard error of the mean. Prototype reference strain H77 is highlighted in *red*.

To ensure reproducibility of neutralization measured using this panel, the panel of 15 HCVpps was reproduced by plasmid transfection at the University of Nottingham, and neutralization testing was repeated with the original 7 reference mAbs (HCV1, hAP33, AR3A, CBH-7, HC84.26, AR4A, and HC33.4). Neutralization results (log_10_IC_50_ values) for each HCVpp and mAb combination obtained at the University of Nottingham were highly correlated with those previously obtained at Johns Hopkins (*r* = 0.92, *P* < .0001) (Figure 6*A*). The mean IC_50_ for each HCVpp across 7 mAbs ranged from 0.63 to >100 μg/mL in Johns Hopkins testing and from 0.74 to 91.38 μg/mL in University of Nottingham testing. None of the HCVpps switched neutralization sensitivity tiers in the repeat testing (Figure 6*B*).

## Discussion

Development of a prophylactic HCV vaccine will require accurate and reproducible measurement of neutralizing breadth and potency of vaccine-induced antibodies. By analyzing hepatoma cell entry, neutralization sensitivity, and neutralization profiles of HCVpps generated with diverse E1E2 clones, we identified a robustly functional and reproducible panel of 15 HCVpps that gave neutralizing potency and breadth measurements for 7 bNAbs that were remarkably similar to results obtained with a more genetically diverse 65-virus panel.

We observed a wide range of neutralization sensitivity across a large number of subtype 1a and 1b HCVpps and a smaller number of genotype 2 to 6 HCVpps. In agreement with prior studies,^17^^,^^28^^,^^29^^,^^33^^,^^48^ the genetic distance between E1E2 clones did not correlate with differences in neutralization sensitivity between HCVpps generated from those clones (Supplementary Figure 5). We also observed very close correlations between neutralization profiles of some HCVpps with very divergent E1E2 sequences, including some close correlations between neutralization profiles of HCVpps from different genotypes. These findings are supported by a recent study by Bankwitz, et al,^48^ which also found that neutralization profiling could be used to select a relatively small number of isolates to represent a larger HCVcc panel. Neutralizing breadth of 4 of the reference mAbs used in this study was previously measured using a widely adopted panel of genotype 1 to 6 HCVccs (strains H77 [gt1a], J6 [gt2a], S52 [gt3a], ED43 [gt4a], SA13 [gt5a], and HK6a [gt6a]),^10^^,^^12^^,^^13^^,^^32^ so it is useful to compare those prior results to the results obtained with this new HCVpp panel. Both panels include some sensitive isolates, but the neutralization breadth of each mAb (defined here as a percentage of isolates in the panel neutralized by ≤10 μg/mL of mAb) was lower using the HCVpp panel than it was in prior studies using the HCVcc panel (Supplementary Table 4), indicating that the HCVpp panel incorporates more antigenic diversity than the HCVcc panel, despite including fewer HCV genotypes. Taken together, these data confirm that the neutralizing breadth of antibodies should be defined by neutralization of antigenically diverse isolates representing multiple tiers of neutralization sensitivity rather than by neutralization of isolates from multiple genotypes.

The neutralization panel described here has some limitations. First, we characterized relatively few genotype 2 to 6 E1E2 clones, and no genotype 7 or 8 clones. However, genotype 2 to 6 HCVpps were distributed among genotype 1 HCVpps in both the neutralization sensitivity and neutralization profile analyses, confirming that neutralization phenotypes do not segregate by genotype or subtype.^34^ In addition, neutralization sensitivities of tier 1 to 4 HCVpps were consistent regardless of the infecting genotype of immune plasma tested. Thus, this reference panel is likely to be antigenically representative of genotype 2 to 8 as well as genotype 1 strains. Nevertheless, it will be important to continue to phenotype additional genotype 2 to 8 isolates.

Second, this panel was generated with HCVpps rather than HCVccs to facilitate widespread use. HCVpps are generally more neutralization sensitive than HCVccs, likely because HCVccs incorporate apolipoprotein E and HCVpps do not.^26^^,^^30^ Therefore, it will be helpful to incorporate some or all of these E1E2 clones into chimeric HCVccs to confirm that relative neutralization sensitivity is consistent across HCVpps and HCVccs, as has been observed in prior studies,^28^, ^29^, ^30^, ^31^

It is interesting that most genotype 4- to 6-infected plasma samples tested with this panel displayed greater neutralizing breadth than genotype 1- to 3-infected plasma. In particular, plasma of genotype 6-infected individuals was very broadly neutralizing. This observation might be the result of different durations of infection at the time of plasma sampling, because longer duration of infection has been associated with greater neutralizing breadth.^17^^,^^49^ Genotype 1 to 3 samples were obtained from the BBAASH acute infection cohort,^40^ with participants infected a median of 389 days (range, 228–963 days) at the time of sampling, whereas genotype 4 to 6 samples were obtained from the University of Nottingham Trent HCV cohort^41^ from individuals with chronic infection of unknown duration. Further studies with time-matched samples from multiple genotypes will be needed to clarify this observation, but these preliminary data suggest that investigation of genotype 6 vaccine antigens for induction of bNAbs may be warranted.

While this panel can be used immediately to compare neutralizing breadth of antibodies induced by candidate vaccines, further work is needed to determine the level of serum neutralizing breadth and neutralizing potency that is necessary for protection against HCV infection. These data might be obtained by using this panel to test serum from humanized mouse models of HCV infection after protective or nonprotective mAb infusions or by measuring plasma neutralizing breadth in vaccine trials performed in at-risk human populations.

As this panel is adopted in new laboratories, quality control of HCVpps will be important. HCVpps should be used in experiments only if they demonstrate entry at least 10-fold above background entry by mockpps. In addition, 1 or more of the reference bNAbs from this study could be included in experiments as a positive control to confirm that neutralization breadth (Table 1) and IC_50_ values (Supplementary Table 2) are similar to those obtained here. Controls for plasma or serum neutralization experiments should also be carefully considered, because we and others have observed enhancement rather than inhibition of infection by some plasma samples. This enhancement of HCVpps entry may be due to lipoproteins in plasma that increase entry when neutralizing antibodies levels are low or absent.^46^^,^^47^ To control for these effects, neutralization by immune plasma or serum should be calculated relative to control wells containing preimmune plasma or serum, or immunoglobulins should be purified from plasma before testing.

In conclusion, we rationally selected and validated a genetically and antigenically diverse panel of 15 HCVpps for use in neutralization assays. This study also demonstrated that neutralizing breadth of HCV antibodies should be defined using viruses spanning multiple tiers of neutralization sensitivity rather than panels selected solely for genetic diversity. We propose that this panel could be adopted as a standard for measurement of antibody neutralizing potency and breadth, advancing HCV vaccine development by facilitating comparisons of neutralization results from laboratories around the world.
