# Supplementary material for: An Antigenically Diverse, Representative Panel of Envelope Glycoproteins for Hepatitis C Virus Vaccine Development
Source: Gastroenterology. 2022 Feb;162(2):562–74. doi: 10.1053/j.gastro.2021.10.005 (PMC8792218; doi:10.1053/j.gastro.2021.10.005)
Supplement: Supplementary Material [file mmc1.pdf]

Supplemental Table 1. E1E2 clone GenBank accession numbers, subtypes, and geographic origin.

E1E2 Tested for Entry

Panel of 75 HCVpp	Accession #	Subtype	Geographic origin
UKNP1.1.1	KU285151	1a	United Kingdom
UKNP1.10.1	KU285172	1a	United Kingdom
UKNP1.11.6	KU285177	1a	United Kingdom
UKNP1.12.2	KU285181	1a	United Kingdom
UKNP1.13.1	KU285182	1a	United Kingdom
UKNP1.14.2	KU285183	1a	United Kingdom
UKNP1.15.1	KU285187	1a	United Kingdom
UKNP1.16.1	KU285190	1a	United Kingdom
UKNP1.16.3	KU285189	1a	United Kingdom
UKNP1.17.1	KU285204	1b	United Kingdom
UKNP1.18.1	KU285192	1b	United Kingdom
UKNP1.19.2	KU285194	1b	United Kingdom
UKNP1.2.1	KU285152	1a	United Kingdom
UKNP1.2.4	KU285155	1a	United Kingdom
UKNP1.2.5	KU285156	1a	United Kingdom
UKNP1.20.4	KU285199	1b	United Kingdom
UKNP1.21.2	KU285202	1b	United Kingdom
UKNP1.21.3	KU285203	1b	United Kingdom
UKNP1.21.4	KU285191	1b	United Kingdom
UKNP1.21.5	KU285205	1b	United Kingdom
UKNP1.21.6	KU285206	1b	United Kingdom
UKNP1.22.1	KU285207	1b	United Kingdom
UKNP1.23.1	KU285208	1b	United Kingdom
UKNP1.3.2	KU285157	1a	United Kingdom
UKNP1.4.3	KU285159	1a	United Kingdom
UKNP1.5.3	KU285165	1a	Pakistan
UKNP1.7.1	KU285167	1a	United Kingdom
UKNP1.8.1	KU285168	1a	United Kingdom
UKNP1.8.2	KU285169	1a	United Kingdom
UKNP1.9.1	KU285170	1a	United Kingdom
1a09	KJ187972	1a	United States
1a115	KY565172	1a	United States
1a116	KM660629	1a	United States
1a123	KJ187980	1a	United States
1a134	KY565177	1a	United States
1a138	KY565180	1a	United States

1a142	KJ187982	1a	United States
1a154 (H77)	KJ187983	1a	United States
1a156	KY565195	1a	United States
1a157	KJ187975	1a	United States
1a18	KY565143	1a	United States
1a35	KY565148	1a	United States
1a38	KJ187974	1a	United States
1a46	KY565154	1a	United States
1a51	KY565155	1a	United States
1a72	KJ187977	1a	United States
1a73	KY565157	1a	United States
1a89	KY565164	1a	United States
1a98	KY565165	1a	United States
1a99	KY565166	1a	United States
1b09	KJ187984	1b	United States
1b14	KJ187985	1b	United States
1b16	KY565207	1b	United States
1b21	KM660628	1b	United States
1b23	KY565209	1b	United States
1b25	KY565210	1b	United States
1b34	KJ187987	1b	United States
1b38	KJ187988	1b	United States
1b39	KY565218	1b	United States
1b43	KY565220	1b	United States
1b45	KY565222	1b	United States
1b52	KJ187989	1b	United States
1b58	KJ187990	1b	United States
UKNP2.2.1	KU285211	2a	United Kingdom
UKNP2.4.1	KU285213	2b	United Kingdom
UKNP3.1.2	KU285215	3a	United Kingdom
UKNP3.2.1	KU285218	3a	United Kingdom
UKNP4.1.1	KU285220	4a	United Kingdom
UKNP4.2.2	KU285222	4a	United Kingdom
UKNP4.3.2	KU285224	4a	United Kingdom
UKNP5.1.1	KU285225	5a	United Kingdom
UKNP5.2.1	KU285226	5a	United Kingdom
UKNP6.1.1	KU285227	6a	Hong Kong
UKNP6.1.2	KU285228	6a	Hong Kong
HK6a	MG717928	6a	n/a

E1E2 Tested for Neutralization

Panel of 65 HCVpp	Accession #	Subtype	Geographic origin
UKNP1.1.1	KU285151	1a	United Kingdom
UKNP1.10.1	KU285172	1a	United Kingdom
UKNP1.11.6	KU285177	1a	United Kingdom
UKNP1.12.2	KU285181	1a	United Kingdom
UKNP1.13.1	KU285182	1a	United Kingdom
UKNP1.14.2	KU285183	1a	United Kingdom
UKNP1.15.1	KU285187	1a	United Kingdom
UKNP1.16.1	KU285190	1a	United Kingdom
UKNP1.16.3	KU285189	1a	United Kingdom
UKNP1.17.1	KU285204	1b	United Kingdom
UKNP1.18.1	KU285192	1b	United Kingdom
UKNP1.19.2	KU285194	1b	United Kingdom
UKNP1.2.4	KU285155	1a	United Kingdom
UKNP1.2.5	KU285156	1a	United Kingdom
UKNP1.20.4	KU285199	1b	United Kingdom
UKNP1.21.2	KU285202	1b	United Kingdom
UKNP1.22.1	KU285207	1b	United Kingdom
UKNP1.23.1	KU285208	1b	United Kingdom
UKNP1.5.3	KU285165	1a	Pakistan
UKNP1.7.1	KU285167	1a	United Kingdom
UKNP1.8.1	KU285168	1a	United Kingdom
UKNP1.8.2	KU285169	1a	United Kingdom
UKNP1.9.1	KU285170	1a	United Kingdom
1a09	KJ187972	1a	United States
1a115	KY565172	1a	United States
1a116	KM660629	1a	United States
1a123	KJ187980	1a	United States
1a134	KY565177	1a	United States
1a138	KY565180	1a	United States
1a142	KJ187982	1a	United States
1a154 (H77)	KJ187983	1a	United States
1a156	KY565195	1a	United States
1a157	KJ187975	1a	United States
1a18	KY565143	1a	United States
1a35	KY565148	1a	United States
1a38	KJ187974	1a	United States
1a46	KY565154	1a	United States
1a51	KY565155	1a	United States
1a72	KJ187977	1a	United States

1a73	KY565157	1a	United States
1a89	KY565164	1a	United States
1a98	KY565165	1a	United States
1a99	KY565166	1a	United States
1b09	KJ187984	1b	United States
1b14	KJ187985	1b	United States
1b16	KY565207	1b	United States
1b21	KM660628	1b	United States
1b23	KY565209	1b	United States
1b25	KY565210	1b	United States
1b34	KJ187987	1b	United States
1b38	KJ187988	1b	United States
1b39	KY565218	1b	United States
1b43	KY565220	1b	United States
1b45	KY565222	1b	United States
1b52	KJ187989	1b	United States
1b58	KJ187990	1b	United States
UKNP2.2.1	KU285211	2a	United Kingdom
UKNP2.4.1	KU285213	2b	United Kingdom
UKNP3.1.2	KU285215	3a	United Kingdom
UKNP3.2.1	KU285218	3a	United Kingdom
UKNP4.1.1	KU285220	4a	United Kingdom
UKNP4.2.2	KU285222	4a	United Kingdom
UKNP5.1.1	KU285225	5a	United Kingdom
UKNP5.2.1	KU285226	5a	United Kingdom
UKNP6.1.1	KU285227	6a	Hong Kong

E1E2 in the Final Panel

Panel of 15 HCVpp	Accession #	Subtype	Geographic origin
UKNP1.10.1	KU285172	1a	United Kingdom
UKNP1.11.6	KU285177	1a	United Kingdom
UKNP1.16.3	KU285189	1a	United Kingdom
UKNP1.18.1	KU285192	1b	United Kingdom
UKNP1.9.1	KU285170	1a	United Kingdom
1a123	KJ187980	1a	United States
1a138	KY565180	1a	United States
1a154 (H77)	KJ187983	1a	United States
1a72	KJ187977	1a	United States
1b25	KY565210	1b	United States
1b34	KJ187987	1b	United States
1b58	KJ187990	1b	United States

UKNP3.1.2	KU285215	3a	United Kingdom
UKNP4.2.2	KU285222	4a	United Kingdom
UKNP5.2.1	KU285226	5a	United Kingdom

Supplemental Table 2. IC_{50} values[1] (µg/mL) for 7 mAbs using the 15 HCVpp panel.

	mAb						
**HCVpp[2]**	**HC33.4**	**HC84.26**	**AR3A**	**AR4A**	**HCV1**	**hAP33**	**CBH7**
**UKNP5.2.1 (5a)**	0.5542	0.9792	0.03515	0.04623	0.1904	0.09503	100
**UKNP1.11.6 (1a)**	0.2372	0.5416	1.133	1.035	0.3677	0.1326	0.6036
**1a154 (1a)**	0.5341	1.022	2.086	3.244	0.859	0.4475	22.97
**1b34 (1b)**	54.35	0.2816	1.122	0.2963	1.743	0.2936	100
**UKNP1.9.1 (1a)**	100	7.931	0.6232	0.4691	0.5916	0.09567	100
**1a123 (1a)**	0.6198	0.4039	100	1.019	0.705	0.7416	100
**1a138 (1a)**	54.29	1.62	1.568	0.7856	0.423	44.2	0.8541
**1b25 (1b)**	10.1	0.9366	9.619	2.06	2.628	1.847	100
**UKNP1.16.3 (1a)**	21.91	100	100	0.3739	1.605	0.7131	14.76
**UKNP4.2.2 (4a)**	6.508	18.17	9.267	3.306	2.579	6.481	100
**1a72 (1a)**	100	4.381	12.36	4.458	7.03	2.799	34.27
**UKNP1.10.1 (1a)**	5.061	100	100	3.534	2.007	2.099	100
**1b58 (1b)**	64.14	5.525	100	24.59	8.976	2.992	100
**UKNP1.18.1 (1b)**	100	100	100	100	100	43.31	100
**UKNP3.1.2 (3a)**	100	100	100	100	100	100	100

[1] HCVpp-mAb combinations that did not reach 50% neutralization were assigned an IC_{50} of 100 µg/mL.
[2] clone name (subtype)

Supplemental Table 3. Percent neutralization of HCVpp by a 1:20 dilution of 35 gt 1-6 plasma samples.

Infecting Genotype	subject	Days Post Infection	Infection Outcome	HCvpp Tier															neutralization breadth (%
				1		2						3					4		
				UKNP5.2.1	UKNP1.11.6	1a123	1a154 (H77)	1b25	UKNP1.9.1	1b34	1a138	UKNP4.2.2	1a72	UKNP1.16.3	UKNP1.10.1	1b58	UKNP3.1.2	UKNP1.18.1	
gt1	1	228	Clearance*	-14	-7	-6	-3	0	21	-12	-13	10	-4	-10	-15	-12	-1	-19	0
	2	339	Clearance	66	85	67	51	51	27	-6	43	31	64	46	18	-18	34	-15	40
	3	376	Clearance	49	66	57	59	54	-20	16	-17	-1	26	-5	19	20	9	45	27
	4	357	Clearance	17	2	-13	11	1	-38	-23	12	-27	-14	-6	-57	-17	-65	-6	0
	5	429	Persistence	76	79	70	65	52	-18	11	51	44	39	-12	31	14	24	1	40
	6	401	Persistence	70	91	50	61	45	-55	-23	22	-27	38	1	-33	-15	-27	-11	20
	7	318	Persistence	13	61	6	13	-1	-83	29	-7	-8	7	26	-7	10	-33	-64	7
	8	363	Persistence	59	76	66	58	15	-72	-14	10	-12	10	-40	-15	6	-18	-33	27
	9	256	Persistence	15	22	9	28	5	-58	-16	-22	-38	-11	12	-21	-2	-65	-35	0
gt2	10	833	Persistence	94	90	71	55	56	28	34	24	58	40	25	10	12	21	-3	40
	11	269	Persistence	87	73	73	45	59	39	35	-62	51	32	15	18	2	3	15	33
	12	730	Persistence	95	86	77	64	39	39	49	13	50	45	24	8	53	30	-7	33
	13	592	Persistence	75	30	12	17	27	3	20	-3	25	4	10	17	0	-5	14	7
	14	572	Persistence	-1	20	2	6	-7	5	18	-62	-11	-7	15	15	-24	-16	9	0
gt3	15	963	Persistence	95	88	70	63	54	43	47	18	50	37	40	22	22	36	8	33
	16	561	Persistence	96	92	59	80	38	41	29	2	35	58	31	23	17	44	28	33
	17	406	Persistence	96	91	75	82	59	35	57	32	50	53	30	39	37	53	27	53
	18	342	Persistence	22	56	22	41	10	11	29	-30	23	14	17	18	10	16	18	7
	19	625	Persistence	80	63	44	42	23	-16	26	-3	30	25	5	13	4	11	7	13
	20	329	Persistence	64	93	57	54	44	12	-7	14	-34	9	8	25	4	10	-29	27
gt4	21	n/a	Persistence	98	96	95	86	65	84	59	94	74	77	73	58	31	13	56	87
	22	n/a	Persistence	99	61	7	33	54	25	41	29	9	25	13	73	-5	2	62	33
	23	n/a	Persistence	100	100	89	84	68	90	59	96	75	55	70	40	36	15	47	73
	24	n/a	Persistence	99	95	76	83	67	81	61	93	90	57	57	54	38	26	55	87
	25	n/a	Persistence	99	96	83	84	53	85	52	93	82	63	74	54	42	23	48	80
gt5	26	n/a	Persistence	97	96	60	69	59	87	42	73	75	48	51	33	5	37	34	60
	27	n/a	Persistence	99	83	78	72	40	80	48	67	49	43	17	17	23	47	-3	40
	28	n/a	Persistence	100	99	94	86	83	92	75	92	79	65	76	65	53	49	73	93
	29	n/a	Persistence	99	97	82	89	88	79	90	98	79	68	38	55	47	29	52	80
	30	n/a	Persistence	11	-1	-1	8	7	63	7	49	12	2	-34	11	-24	0	-3	7
gt6	31	n/a	Persistence	99	95	93	99	97	98	100	97	95	94	97	94	86	79	100	100
	32	n/a	Persistence	99	98	80	92	86	93	97	93	91	87	87	92	92	77	88	100
	33	n/a	Persistence	98	90	78	88	84	87	92	87	89	91	93	89	73	82	94	100
	34	n/a	Persistence	100	99	82	94	96	85	95	92	93	90	99	92	80	67	94	100
	35	n/a	Persistence	100	94	73	97	92	87	95	100	98	95	97	95	74	82	93	100

*plasma isolated 1 month after clearance of infection

Supplemental Table 4. Comparison of neutralizing breadth of reference mAbs measured using genotype 1-6 HCVcc (published data) or the panel of 15 HCVpp.

mAb	No. (%) of genotype 1-6 HCVcc neutralized[1]	No. (%) of HCVpp panel neutralized[1]	Source of HCVcc data
HC33.4	3 of 6 (50)	6 of 15 (40)	(Keck, J. Virol., 2013)
AR3A	5 of 6 (83)	8 of 15 (53)	(Giang, PNAS, 2012)
AR4A	6 of 6 (100)	12 of 15 (80)	(Giang, PNAS, 2012)
HC84.26	4 of 5 (80)[2]	10 of 15 (67)	(Keck, PlosPath, 2012)

[1]Number of isolates neutralized at an IC_{50} threshold of 10 μg/mL (i.e., number of isolates neutralized >50% at a mAb concentration ≤10 μg/mL).
[2]Genotype 6 HCVcc was not tested for this mAb.

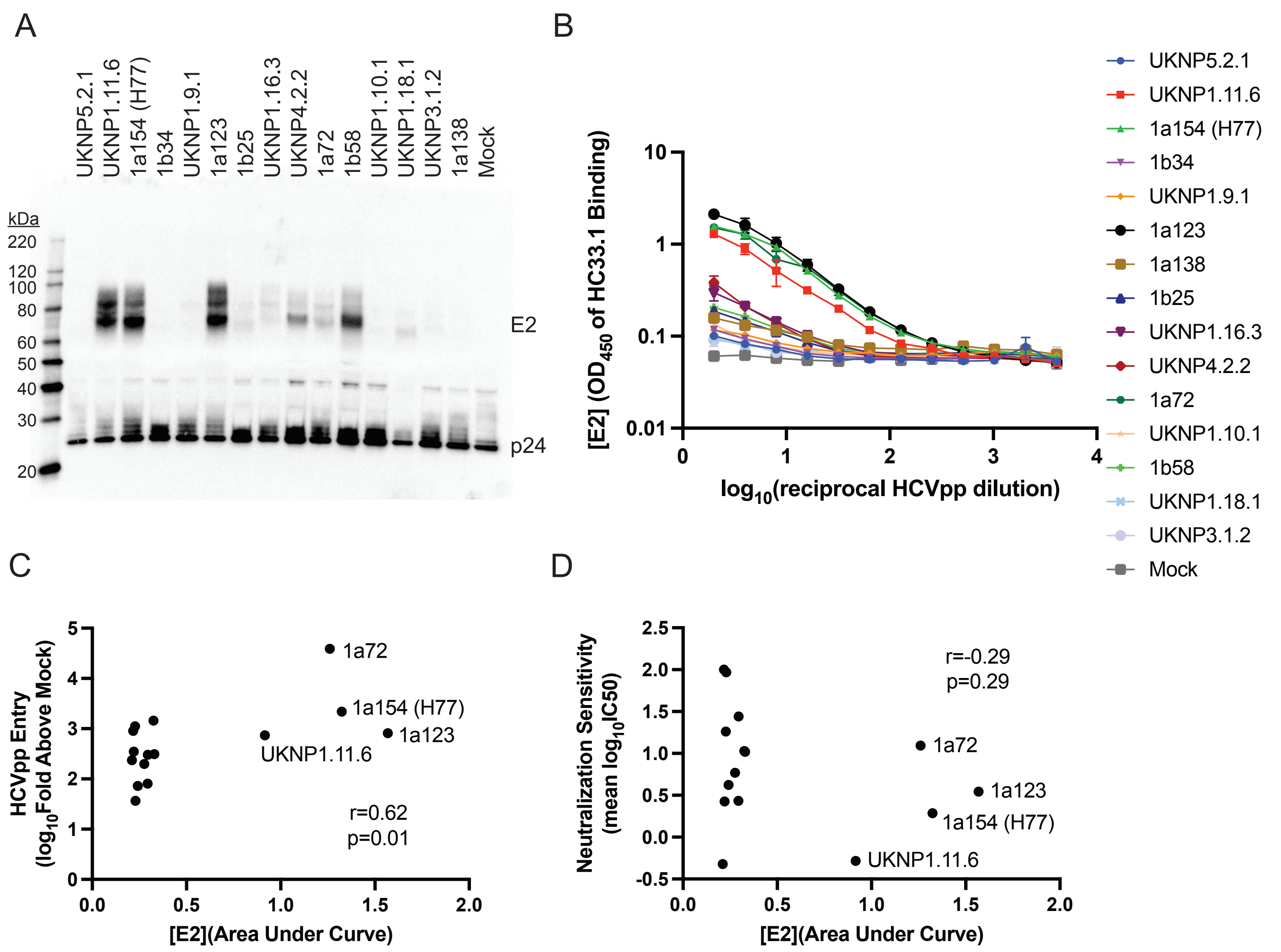

Supplemental Figure 1. Quantitation of E2 incorporated into HCVpp. A. Western blot of a denaturing, reducing gel run with concentrated HCVpp, probed with an anti-E2 mAb (HC33.1) targeting a linear epitope that is intact in all E2 variants and an anti-HIV p24 mAb. **B.** ELISA with serial 2-fold dilutions of concentrated, denatured HCVpp bound to GNA-lectin-coated wells, probed with anti-E2 (HC33.1). Values are means of duplicate wells, and error bars are SEM. **C.** Positive correlation of the amount of E2 incorporated into each HCVpp (area under the curve calculated in B) with entry of that HCVpp into Huh7 hepatoma cells (expressed as fold entry above background entry of mock HCVpp with no E1E2). **D.** No correlation of the amount of E2 incorporated into each HCVpp (area under the curve calculated in B) with neutralization sensitivity of that HCVpp (mean $\log_{10}IC_{50}$ for each HCVpp from Figure 2). R and p values were calculated using the Pearson method, with $p<0.05$ considered significant.

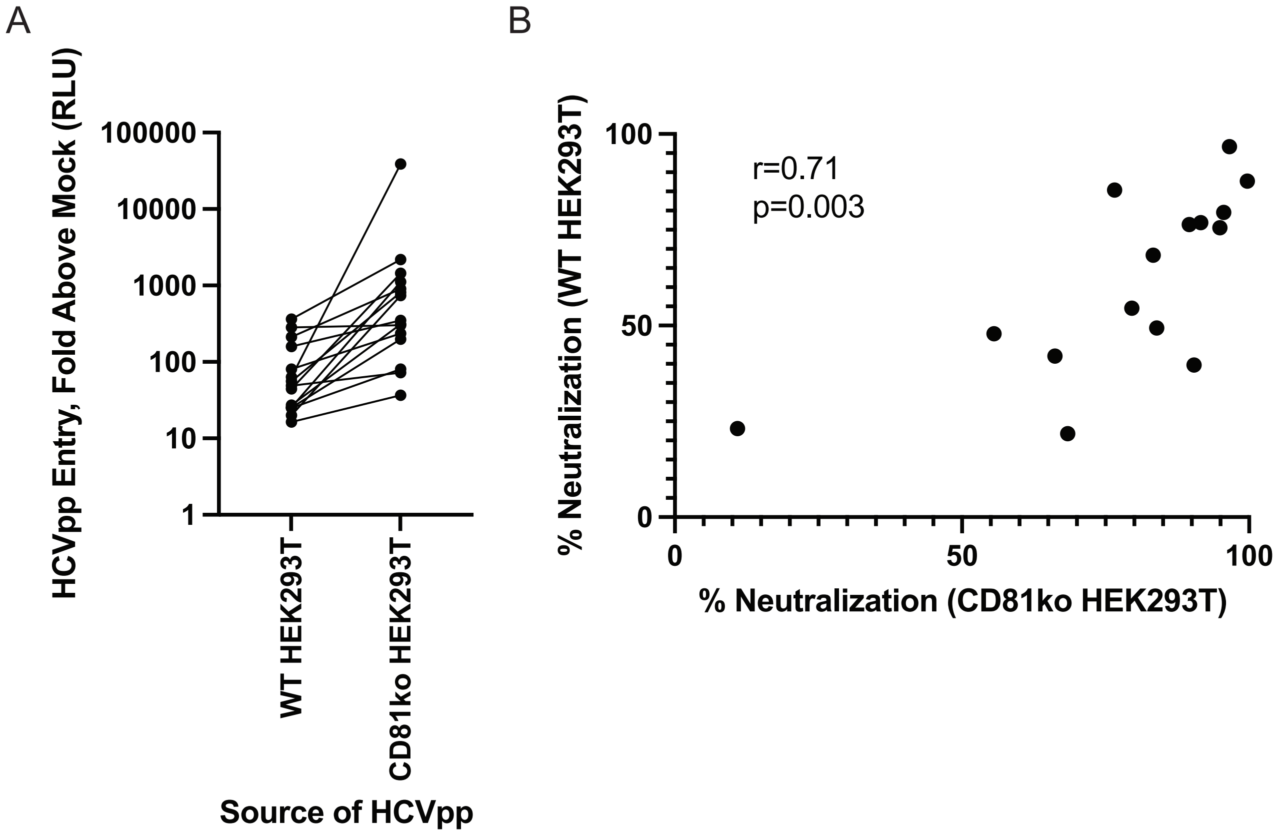

Supplemental Figure 2. A. Hepatoma cell (Huh7) entry of 15 HCVpp produced by transfection of either wild type (WT) HEK293T cells or CD81 knockout (CD81ko) HEK293T cells, expressed as fold increase in RLU above background entry of mock HCVpp without E1E2 transfected and tested in parallel. **B.** Correlation between percent neutralization values obtained using mAb HEPC74 at 20 μg/mL concentration and 15 HCVpp produced in WT HEK293T cells or CD81ko HEK293T cells. R and p values were calculated using Pearson correlation.

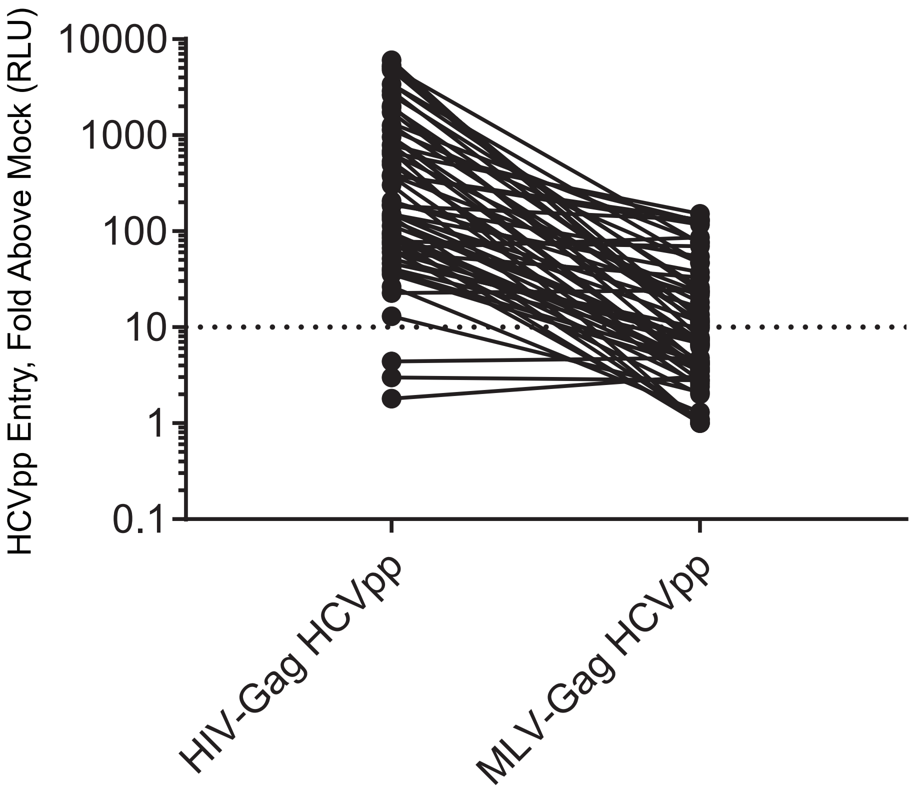

Supplemental Figure 3. Initial testing to compare specific entry of HIV-Gag HCVpp to MLV-Gag HCVpp. Sixty genotype 1a or 1b E1E2 were co-transfected with either *env*-deficient HIV-1 with a luciferase reporter gene (HIV-Gag HCVpp) or with a luciferase-encoding reporter plasmid and an MLV Gag/Pol packaging construct (MLV-Gag HCVpp). Mockpp (lacking E1E2) were produced in parallel, and entry was calculated as a ratio of HCVpp entry relative to mockpp entry. The pre-selected threshold of 10-fold greater than mockpp entry is indicated with a dotted line.

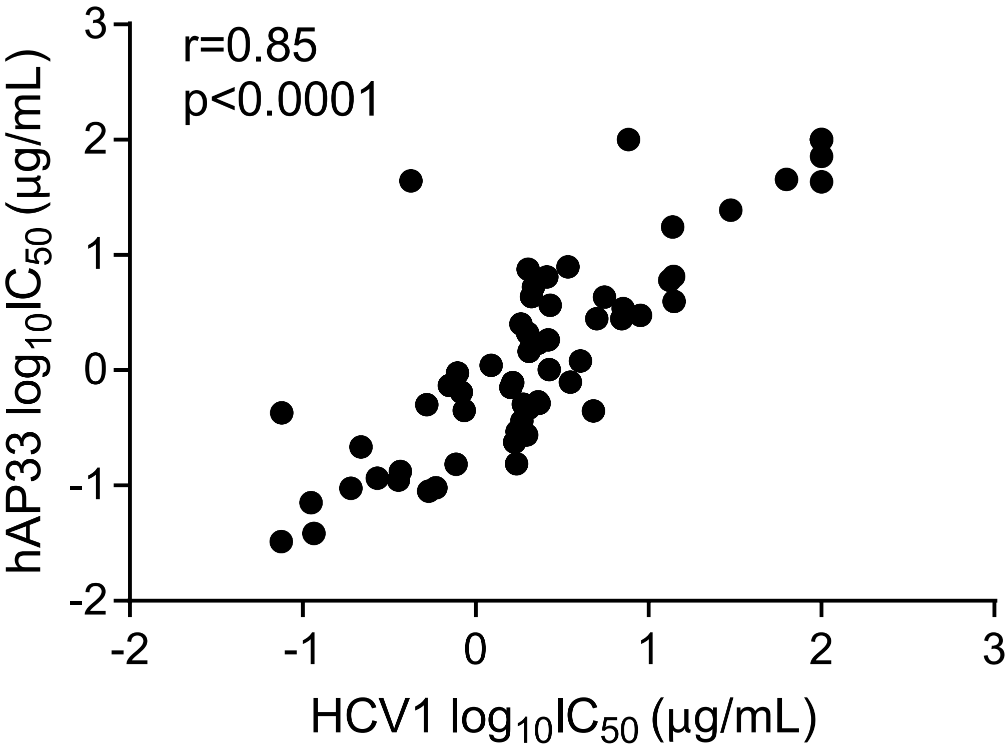

Supplemental Figure 4. Correlation between IC_{50} values across 65 HCVpp for bNAbs HCV1 and hAP33. Given the similarity of epitopes targeted by these bNAbs, we measured the correlation between $\log_{10}IC_{50}$ values for these mAbs across the HCVpp panel. Due to this high correlation in neutralization profiles, $\log_{10}IC_{50}$ values for these two mAbs for each HCVpp were averaged (given half the weight of the other 5 bNAbs) when ranking HCVpp by neutralization sensitivity and for hierarchical clustering of HCVpp (Figures 2-4, 6). R and p calculated by the Pearson method.

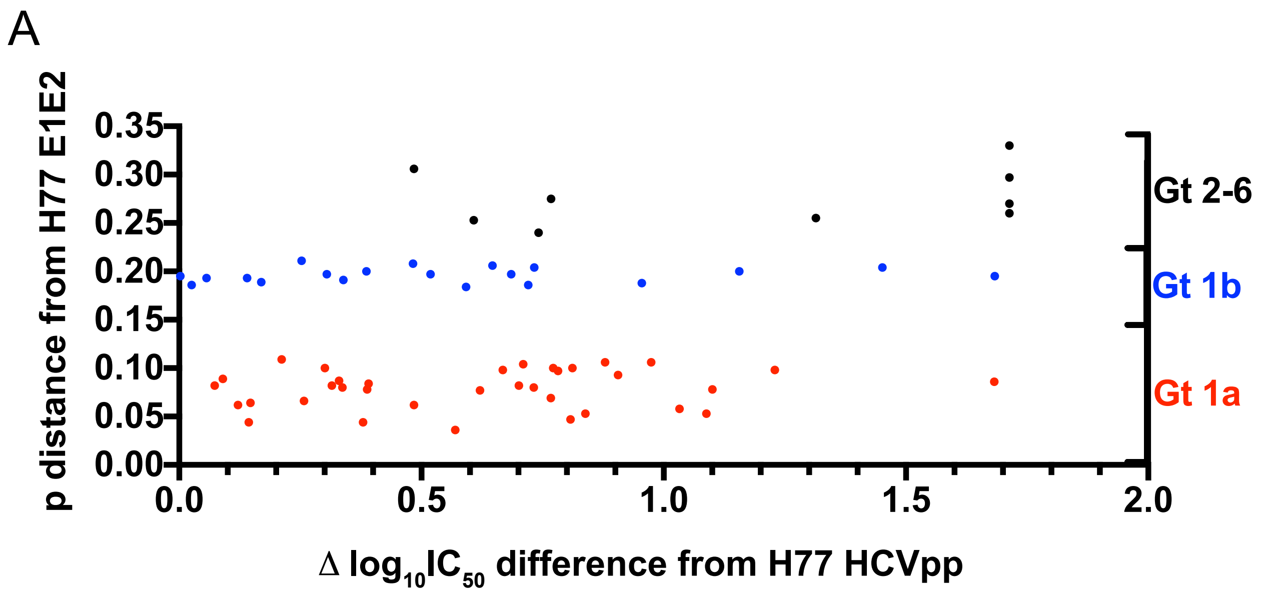

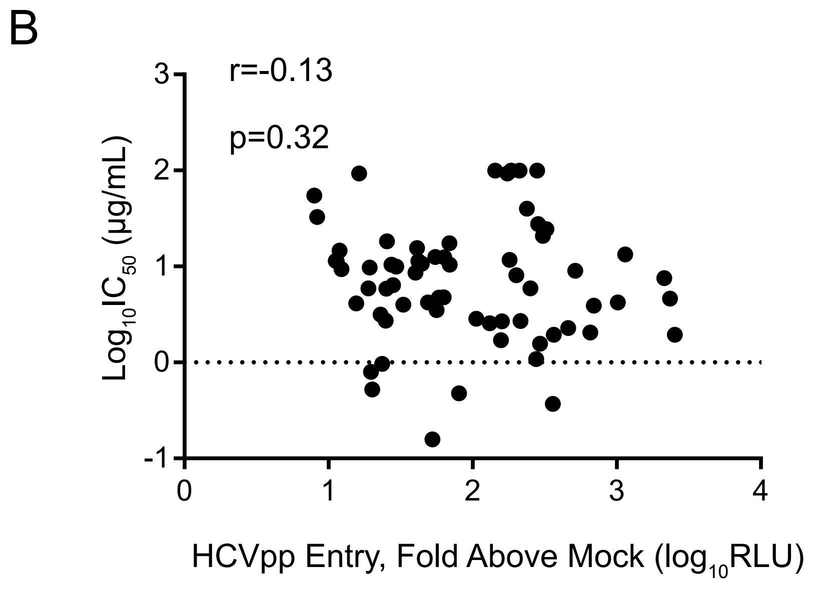

Supplemental Figure 5. Genetic distance and magnitude of hepatoma cell entry do not predict neutralization sensitivity of HCVpp. A. For each of 64 HCVpp, E1E2 amino acid p distance and absolute difference in mean $\log_{10}IC_{50}$ were calculated relative to reference strain 1a154 (H77, genotype 1a) HCVpp. The expected genetic distances were observed between H77 and genotype 1a (red), genotype 1b (blue), and genotype 2-6 (black) E1E2 clones, but this distance was not correlated with the difference in neutralization sensitivity of HCVpp generated with these clones relative to H77 HCVpp sensitivity. **B.** No correlation between magnitude of hepatoma cell entry (Figure 2A) and mean $\log_{10}IC_{50}$ (Figure 2B) of 65 HCVpp. R and p values calculated using the Spearman method.

A

mAbs	Tier 1		Tier 2						Tier 3					Tier 4	
HCVpp	UKNP5.2.1	UKNP1.11.6	1a154	1a123	1a138	1b25	1b34	UKNP1.9.1	1a72	UKNP1.16.3	UKNP4.2.2	1b58	UKNP1.10.1	UKNP1.18.1	UKNP3.1.2
HEPC74	87	96	73	74	79	80	48	81	72	40	40	36	24	66	40
HEPC108	83	88	83	83	83	65	51	52	59	39	39	15	44	42	63
HC33.1	86	72	69	80	74	78	23	13	50	43	17	29	33	64	28
HEPC146	30	60	54	78	50	42	8	9	54	40	32	37	32	-12	22
HEPC111	59	67	66	61	70	32	3	-1	47	25	-1	8	31	41	8
HEPC98	-3	-36	99	-2	-1	5	-10	33	21	-5	82	3	-34	31	-44
HEPC112	26	-26	50	19	22	14	6	-12	46	-103	10	12	10	6	-1

% Neut.
>75
>50
>25
<25

B

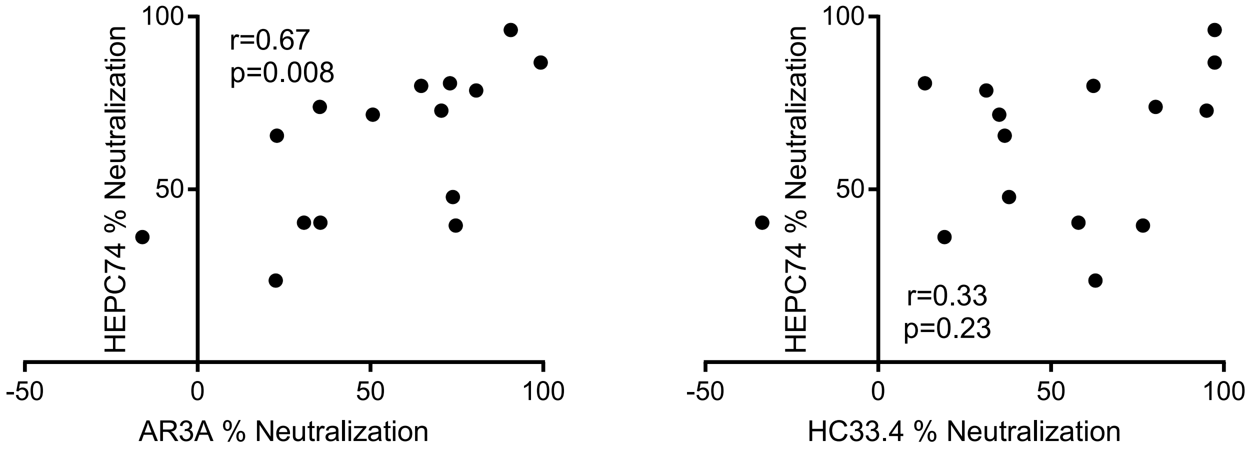

Supplemental Figure 6. A. % neutralization of the 15 HCVpp panel by 7 additional neutralizing mAbs at 20 μg/mL concentration, measured in duplicate. **B.** Correlation between % neutralization values obtained with HEPC74 and related mAb AR3A, or HEPC74 and unrelated mAb HC33.4, tested at 20 μg/mL concentration. Each point represents neutralization of a single HCVpp measured in duplicate. R and p values determined using the Spearman method.
